# Oxidative Stress and Antimicrobial Activity of Chromium(III) and Ruthenium(II) Complexes on *Staphylococcus aureus* and *Escherichia coli*

**DOI:** 10.1155/2013/906912

**Published:** 2013-09-04

**Authors:** Paulina L. Páez, Claudia M. Bazán, María E. Bongiovanni, Judith Toneatto, Inés Albesa, María C. Becerra, Gerardo A. Argüello

**Affiliations:** ^1^Departamento de Farmacia, Facultad de Ciencias Químicas, Universidad Nacional de Córdoba, X5000HUA Córdoba, Argentina; ^2^Instituto de Investigaciones en Físico Química de Córdoba (INFIQC) CONICET-UNC, Departamento de Físico Química, Facultad de Ciencias Químicas, Universidad Nacional de Córdoba, Ciudad Universitaria, X5000HUA Córdoba, Argentina; ^3^IMBIV-CONICET, Instituto Multidisciplinario de Biología Vegetal, Ciudad Universitaria, X5000HUA Córdoba, Argentina

## Abstract

The prevalence of antibiotic resistance has resulted in the need for new approaches to be developed to combat previously easily treatable infections. The main aim of this work was to establish the potential of the synthetic **α**-diimine chromium(III) and ruthenium(II) complexes (where the **α**-diimine ligands are bpy = 2,2-bipyridine, phen = 1,10-phenanthroline, and dppz = dipyrido[3,2-a:2′,3′-c]-phenazine) like [Cr(phen)_3_]^3+^, [Cr(phen)_2_(dppz)]^3+^, [Ru(phen)_3_]^2+^, and [Ru(bpy)_3_]^2+^ as antibacterial agents by generating oxidative stress. The [Cr(phen)_3_]^3+^ and [Cr(phen)_2_(dppz)]^3+^ complexes showed activity against Gram positive and Gram negative bacteria with minimum inhibitory concentrations (MICs) ranging from 0.125 **μ**g/mL to 1 **μ**g/mL, while [Ru(phen)_3_]^2+^ and [Ru(bpy)_3_]^2+^ do not exhibit antimicrobial activity against the two bacterial genera studied at the concentration range used. When ciprofloxacin was combined with [Cr(phen)_3_]^3+^ for the inhibition of *Staphylococcus aureus* and *Escherichia coli*, an important synergistic effect was observed, FIC 0.066 for *S. aureus* and FIC 0.064 for *E. coli*. The work described here shows that chromium(III) complexes are bactericidal for *S. aureus* and *E. coli*. Our results indicate that **α**-diimine chromium(III) complexes may be interesting to open new paths for metallodrug chemotherapy against different bacterial genera since some of these complexes have been found to exhibit remarkable antibacterial activities.

## 1. Introduction

Since their discovery, antibiotics have played an important role in health care. However, the increasing emergence of antibiotic resistance among a variety of microbial pathogens stimulates intensive research efforts with the aim of identifying alternative therapeutic approaches [1]. 

The chemistry of metallic complexes with heterocyclic ligands has attracted the interest of both inorganic and bioinorganic chemists in recent years, and the field of coordination chemistry of metallic complexes has undergone a remarkable growth during the past few decades and became a growing class of research [2]. This enormous growth is attributed to the synthesis of a large number and variety of synthetic ligands (chelates) which behave as coordinating agents for metal ions [3]. It is known that 2,2′-bipyridine and 1,10-phenanthroline chelators act as potential antitumor agents [4, 5] and several metal chelates possess antibacterial, antifungal, antiviral and anticancer activities. However, in several cases, it has been found that metal chelates have more antimicrobial activity than the chelating agents themselves [6].

 The study of the chromium complexes in coordination chemistry is of great significance due to their various applications in biological processes. In vitro studies have demonstrated that chromium in the proper ligand environment can lead to DNA damage, plasmid cleavage, and protein cleavage [7–9]. Some reports showed chromium complexes as the cause of oxidation of DNA by binding with guanine [10, 11]. Additionally, octahedral transition metal complexes containing three bipyridine or phenanthroline ligands have been shown to be groove binders or possible partial DNA intercalators [12, 13], whereas some chromium(III) Schiff base complexes prefer nonintercalative mode of binding to DNA [7]. On the other hand, it has been reported that the bovine serum albumin (BSA) protein damage brought about by chromium(III) complexes depends on the nature of the coordinated ligand and the nature of the metal complex [9].

As part of an ongoing program to investigate the interaction of transition metal with double-helical DNA, we found that the *α*-diimine chromium(III) complexes are associated with DNA by noncovalent interactions. The chromium(III) complexes are capable of producing plasmidic DNA photodamage with high efficiency of photocleavage [14–16]. This leads to consider these complexes as attractive photosensitizing agents to be used in photodynamic therapy (PDT).

On the other hand, coordination complexes of transition metals have been widely studied due to their antimicrobial properties [17–21]. The importance of chromium as an environmental pollutant and its involvement in the toxicity in animals and plants have made the study of the interactions of derivatives of this metal with microbial cells in a fertile field for both basic research and applied science [22]. In this sense, it was reported that chromium(III) complexes containing oxalohydrazide and quinoxaline as ligands would have microbiological activity [23, 24], reducing virulence and antibiotic resistance, causing loss of the plasmid of *Shigella dysenteriae* that contains genes encoding virulence factors responsible for this organism's ability to penetrate and multiply into the cells [25]. Furthermore, it has been reported that chromium(III) complexes containing amino acids as ligands have antibacterial and antifungal activities [26], and the authors suggest that the mechanism of antibacterial activity of this complexes would be due to oxidative DNA damage. Emerging reports on the biotoxicity of chromium(III) complexes have emphasized the effects of the coordinated ligand on metal activity [27].

Diverse substances affect the reactive oxygen species (ROS) generation in bacterial cells, and they have the capacity to undergo redox cycling, resulting in the generation of superoxide anion (O_2_^−^) and hydrogen peroxide (H_2_O_2_). We have described that several antibiotics induce oxidative stress in bacteria, among them ciprofloxacin [28–31]. 

The aim of this investigation was to study the potential antimicrobial activity of certain chromium(III) and ruthenium(II) complexes and their combination with ciprofloxacin, and this will provide an understanding of the potential enhancing of the activity of this antibiotic by generating oxidative stress in two different bacterial genera. 

## 2. Materials and Methods

### 2.1. Reagents

1,10-Phenanthroline (phen), tris(1,10-phenanthroline)ruthenium(II) chloride ([Ru(phen)_3_]Cl_2_), and tris(2,2′-bipyridine)ruthenium(II) chloride ([Ru(bpy)_3_]Cl_2_) were purchased from Sigma and used as received. Tris-phenanthroline chromium (III) perchlorate ([Cr(phen)_3_](ClO_4_)_3_) was obtained from previous studies, synthesized according to the literature, and recrystallized at least three times. The ligand dppz was synthesized and purified according to the literature [32]. Bis(1,10-phenanthroline)dipyrido[3,2-a:2′,3′-c]-phenazine chromium(III) triflate ([Cr(phen)_2_(dppz)](CF_3_SO_3_)_3_) was synthesized with slight modifications according to a previously reported procedure [33]. The stock solutions [Cr(phen)_3_]^3+^ and [Cr(phen)_2_(dppz)]^3+^ were prepared in phosphate buffer saline (PBS, pH 7.4), and the concentration of chromium complexes was calculated using molar extinction coefficients of *ε*_354_ = 4200 M^-1 ^cm^−1^ and *ε*_360_ = 13900 M^-1 ^cm^−1^, respectively [33]. CrCl_3_ · 6H_2_O (Fluka, purity 98%) was used without further purification. Millipore Milli Q water was used for preparing buffer solutions. All other chemical reagents were of analytical grade. 

### 2.2. Minimal Inhibitory Concentration (MIC) Determination

The antimicrobial activity of the compounds in *Staphylococcus aureus* ATCC 29213 and *Escherichia coli* ATCC 25922 was evaluated by using the standard tube dilution method on Mueller Hinton broth (MH, Britania). Strains coming from cultures of 24 h in Mueller Hinton medium were diluted to 10^6^ colony-forming units (CFU) per mL and incubated for 10 min at 37°C; the compounds were then added at different concentrations (from 0.004 to 2 *μ*g/mL). Bacterial growth was observed at 18 h of incubation, following the indications of the Clinical and Laboratory Standards Institute [34]. For comparison, chromium(III) and ruthenium(II) complexes were tested. In addition, the MICs of the ligands (bpy, phen and dppz) and CrCl_3_ · 6H_2_O were calculated. Serial dilutions in phosphate buffer solution (PBS), pH 7.4 of the complexes (0.5 mL), were made. The concentrations of the compounds were ranged from 0.004 to 2 *μ*g/mL. Dilutions of a standard antibiotic (from 0.008 to 4 *μ*g/mL of ciprofloxacin) were also prepared in the same manner. An overnight culture of each microorganism was diluted to achieve a cell density in the range from 10^5^ to 10^7^ CFU/mL. The cell suspension (0.5 mL) was inoculated into each tube to give a total volume of 1 mL. The lowest concentration of each complex that prevented bacterial growth was considered to be the MIC. Inoculated complexes-free broths were used as negative controls. Viable bacterial counts were obtained for control samples by plating serial dilutions on MH agar plates, followed by aerobic incubation at 37°C for 18 h for *E*. *coli *and *S. aureus*.

### 2.3. Time-Kill Study

The bactericidal activity was explored by the time-kill method according to CLSI [34]. Kill curves were constructed by plotting the CFU/mL surviving at each time point in the presence and absence of [Cr(phen)_3_]^3+^ in *E. coli* ATCC 25922 and *S. aureus* ATCC 29213.

### 2.4. Checkerboard Assay

The interactions between the chromium(III) complexes and ciprofloxacin were evaluated by the checkerboard method [35]. Bacterial growth inhibition resulting from the interactions was determined by the microdilution test. The concentrations of each tested agent used in the combinations corresponded to serial 2-fold dilutions from their MIC values. The fractional inhibitory concentration (FIC) was calculated using the MIC from the checkerboard assay and the MIC of each compound alone, obtained in parallel in the same assay, according to the following formula: FIC = MIC of antimicrobial agent in combination/MIC of antimicrobial agent alone. Then, the synergistic effect was evaluated by calculating the FICindex (FICI) for each combination, by adding the individual FIC values. An FICI of 0.5 indicated synergy for the combination. When it fell between 0.5 and 1, it was defined as an additive effect, and between 1.0 and 4.0 it was classified as “no interaction.” Finally, an FICI > 4.0 indicated antagonism between the components in the combination.

### 2.5. Determination of ROS by Chemiluminescence (CL) Assay

Oxidative stress of *S. aureus* ATCC 29213 *and E. coli *ATCC 25922 was investigated in a BioOrbit luminometer. Bacteria were cultured in triptein soy broth (TSB) at 37°C for 24 h. 0.1 mL of bacterial suspension was incubated with 0.1 mL of 75 **μ**g/mL lucigenin (bis-N-methylacridinium dinitrate), 0.1 mL of PBS, pH 7.2 and 0.1 mL of ciprofloxacin (from 0.033 to 4 **μ**g/mL) and 0.1 mL of [Cr(phen)_3_]^3+^ (from 0.033 to 4 **μ**g/mL). Finally, the reaction was triggered with 0.1 mL of dimethylsulfoxide (DMSO) in the moment of determination. Controls of basal production of ROS were performed with bacteria in absence of antibiotic. The light emitted by ROS was expressed as relative light unities (RLU) at different times in seconds.

## 3. Results 

### 3.1. Minimal Inhibitory Concentration (MIC) Determination

**Figure 1:**
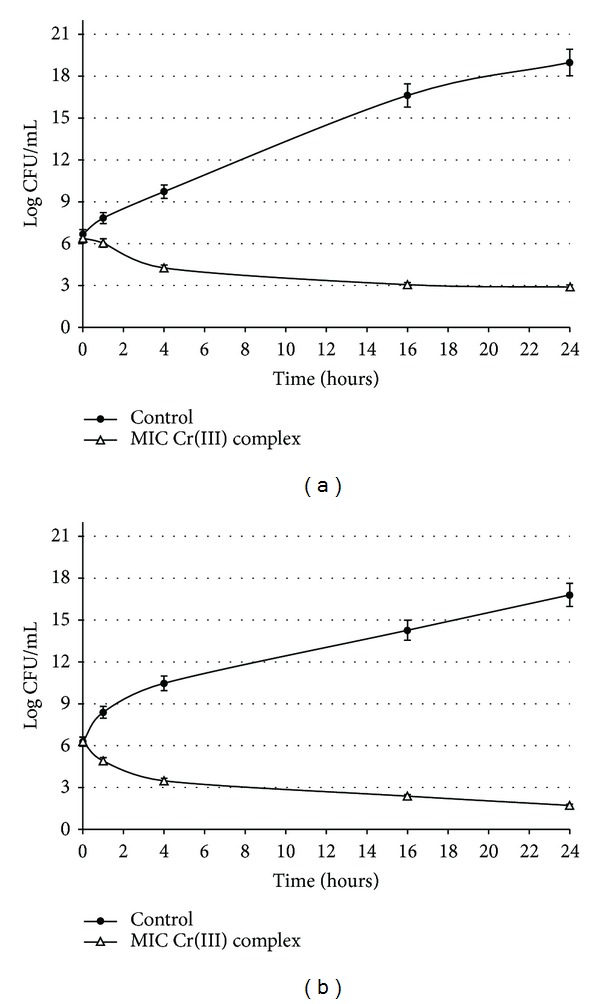
Time-kill kinetics of [Cr(phen)_3_]^3+^ complex at minimum inhibitory concentration (MIC) (Δ) and control (•). (a) *Staphylococcus aureus* ATCC 29213. (b) *Escherichia coli* ATCC 25922.

Four chemically macrocyclic complexes and their ligands (Figure 1) were screened for their *in vitro* antibacterial activity against two bacteria, the Gram positive bacterium *S. aureus *and the Gram negative bacterium *E. coli*. Despite the apparent similarities of the compounds, the preliminary experiments showed that they present different antimicrobial activities.

Chromium(III) complexes demonstrated antimicrobial activity against Gram positive and Gram negative bacteria. The Ru(II) complexes showed less activity than the Cr(III) complexes (Table 1). The bpy, phen, dppz, and CrCl_3_ · 6H_2_O (Cr^3+^ as free ion) show poor activity in comparison with that of the metal complexes against the two bacterial strains. Among all complexes of the series under test for determination of MIC (Figure 1), [Cr(phen)_2_(dppz)]^3+^ was found to be the most potent. It registered MIC as shown by ciprofloxacin against two bacterial strains. 

*E. coli* was found to be more susceptible to [Cr(phen)_2_(dppz)]^3+^ than *S. aureus*, with an MIC for *E. coli *of 0.125 *μ*g/mL and an MIC for *S. aureus *of 0.5 *μ*g/mL while the MIC for *E. coli *was 0.25 *μ*g/mL and for *S. aureus *1 *μ*g/mL for [Cr(phen)_3_]^3+^. Unlike those found for the ligands or Cr^3+^ alone, synthesized metal complexes of chromium(III) showed high antimicrobial activities against *E. coli* and *S. aureus.*

### 3.2. Checkerboard Assay

**Figure 2:**
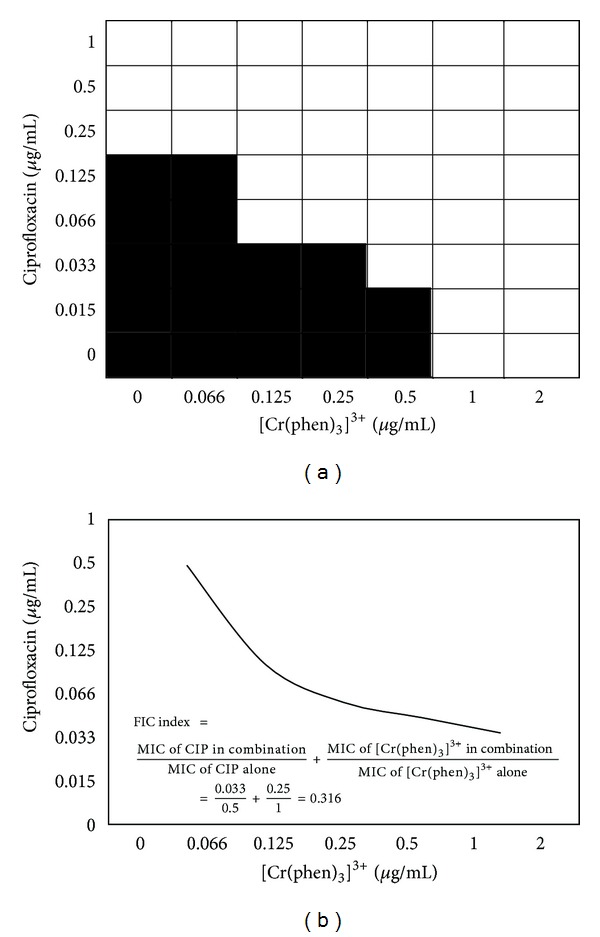
The fractional inhibitory concentration (FIC) index of [Cr(phen)_3_]^3+^ in *Staphylococcus aureus *ATCC 29213 using the checkerboard technique.

**Figure 3:**
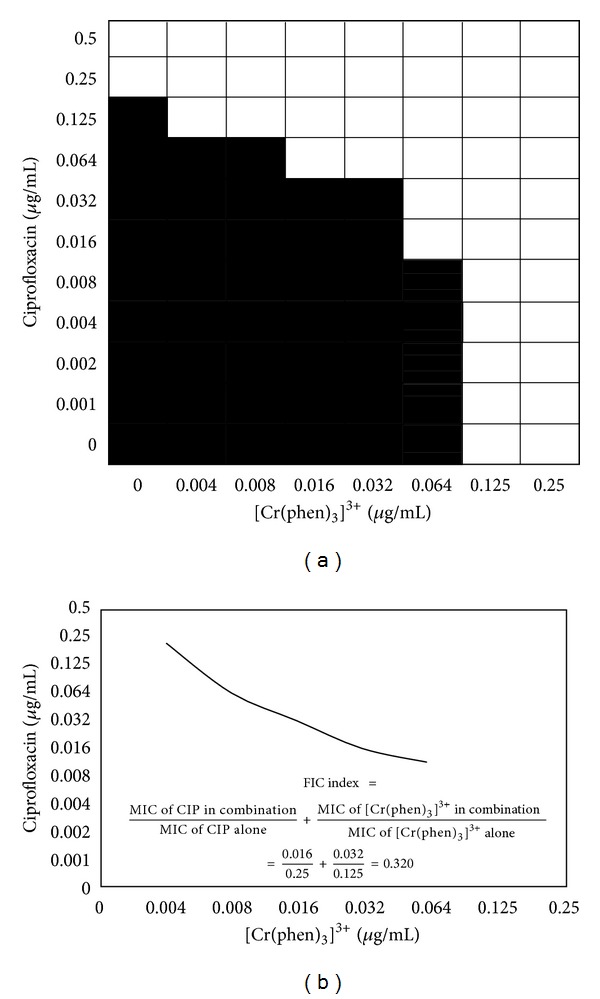
The fractional inhibitory concentration (FIC) index of [Cr(phen)_3_]^3+^ in *Escherichia coli *ATCC 25922 using the checkerboard technique.

When ciprofloxacin was combined with [Cr(phen)_3_]^3+^ for the inhibition of *S. aureus* and *E. coli*, an important synergistic effect was observed (Tables 2 and 3). A notable synergism was observed in *E. coli* (FIC 0.032) when the concentration of the ciprofloxacin was decreased to 0.016 *μ*g/mL, equivalent to 15 times less than its MIC (Table 3). When the [Cr(phen)_3_]^3+^ was combined with ciprofloxacin, a surprising synergistic effect (FICI_C+A_ = 0.096) was observed, even when the concentration of the former was decreased 4 times below its MIC and of the antibiotic 31 times below its individual MIC (Figures 2 and 3).

### 3.3. Time-Kill Study

When [Cr(phen)_3_]^3+^ was tested against *S. aureus* (Figure 1) at MIC, a difference of 3 log_10_ in the number of viable cells compared to the control was observed after 16 h of incubation. When this complex was assayed against *E. coli*, the treatment at MIC reached differences of 3 log_10_ with respect to the control after 8 h of exposure. At this time, a complete bactericidal effect was observed. 

### 3.4. Determination of ROS by Chemiluminescence (CL) Assay

**Figure 4:**
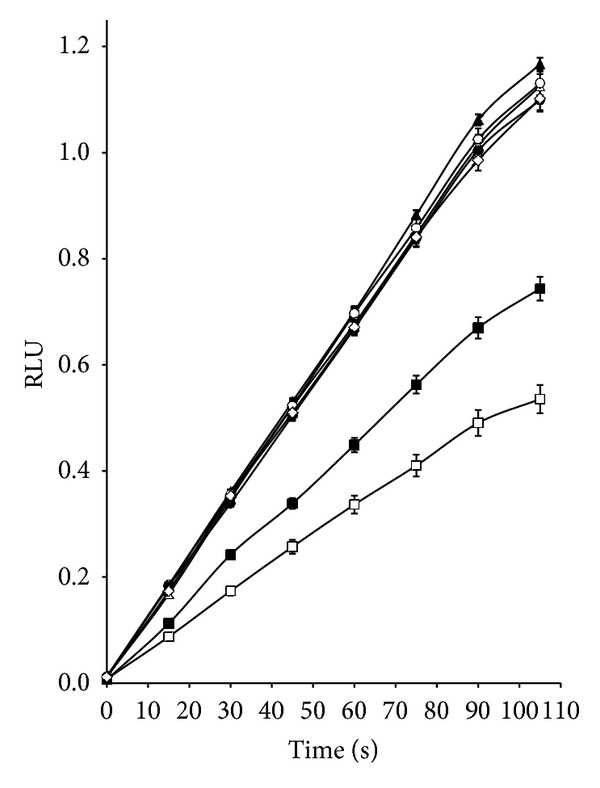
ROS generation in *Staphylococcus aureus* ATCC 29213 incubated with [Cr(phen)_3_]^3+^ at MIC concentration determined by chemiluminescence assay. Control (□); treated with 1 *μ*g/mL [Cr(phen)_3_]^3+^ and 0.125 *μ*g/mL ciprofloxacin (■); treated with 1 *μ*g/mL [Cr(phen)_3_]^3+^ and 0.25 *μ*g/mL ciprofloxacin (Δ); treated with 1 *μ*g/mL [Cr(phen)_3_]^3+^ and 0.5 *μ*g/mL ciprofloxacin (▲); treated with 1 *μ*g/mL [Cr(phen)_3_]^3+^ and 1 *μ*g/mL ciprofloxacin (*◦*); treated with 1 *μ*g/mL [Cr(phen)_3_]^3+^ and 2 *μ*g/mL ciprofloxacin (•); treated with 1 *μ*g/mL [Cr(phen)_3_]^3+^ and 4 *μ*g/mL ciprofloxacin (*◊*).

Chemiluminescence assays indicated a higher degree of oxidative stress in *S. aureus* ATCC 29213 when it was treated with a combination of [Cr(phen)_3_]^3+^ at 1 *μ*g/mL with ciprofloxacin at 0.5 *μ*g/mL, and the maximum value of RLU obtained was 1.167 ± 0.016, representing an increase of 2-fold with respect to the control without antibiotic or the complex (Figure 4), while for the combination of [Cr(phen)_3_]^3+^ at 4 *μ*g/mL (concentration supra MIC) with ciprofloxacin at 0.5 *μ*g/mL, the increase in RLU was 3.9-fold with respect to the control (data not shown).

**Figure 5:**
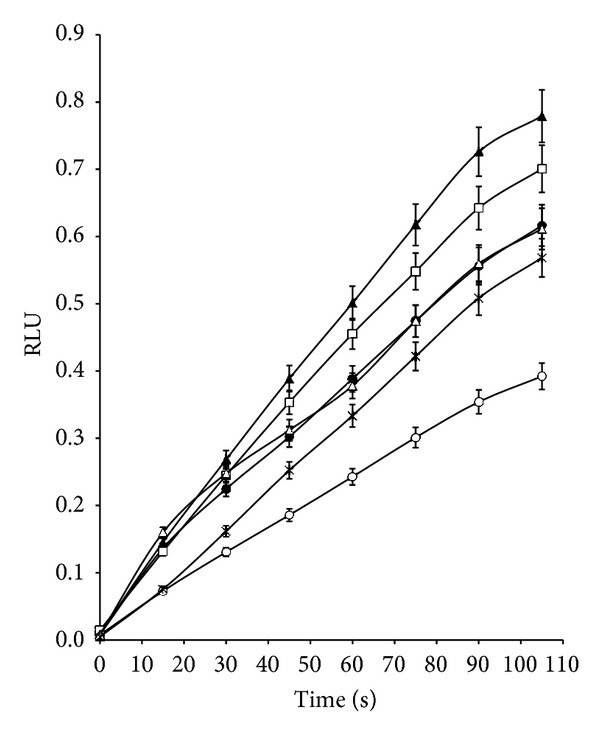
ROS generation in *Escherichia coli* ATCC 25922 incubated with [Cr(phen)_3_]^3+^ at MIC concentration determined by chemiluminescence assay. Control (*◦*); treated with 0.125 *μ*g/mL [Cr(phen)_3_]^3+^ and 0.06 *μ*g/mL ciprofloxacin (•); treated with 0.125 *μ*g/mL [Cr(phen)_3_]^3+^ and 0.125 *μ*g/mL ciprofloxacin (□); treated with 0.125 *μ*g/mL [Cr(phen)_3_]^3+^ and 0.5 *μ*g/mL ciprofloxacin (▲); treated with 0.125 *μ*g/mL [Cr(phen)_3_]^3+^ and 1 *μ*g/mL ciprofloxacin (Δ); treated with 0.125 *μ*g/mL [Cr(phen)_3_]^3+^ and 2 *μ*g/mL ciprofloxacin (*∗*).

When *E. coli* was treated with a combination of [Cr(phen)_3_]^3+^ at 0.125 *μ*g/mL with ciprofloxacin at 0.5 *μ*g/mL, the maximum value of RLU obtained was 0.779 ± 0.040, representing an increase of 2-fold with respect to the control (Figure 5), while for the combination with lower concentrations of [Cr(phen)_3_]^3+^ and ciprofloxacin, 0.03 and 0.06 *μ*g/mL, respectively, the increase of RLU with respect to the control was 4.8-fold.

These results were in agreement with the level of susceptibility of each strain to the combination of the complex with the antibiotic, since *S. aureus* was more resistant than *E. coli, *and this could have been related to less generation of ROS occurring in the Gram positive bacteria.

## 4. Discussion

Despite the range of antibacterial agents currently available, the development of new therapeutic alternatives to deal with pathogens is of great interest for researchers and pharmaceutical industries. In our biological experiments, by using chromium(III) complexes we have observed high biological activity against Gram negative and Gram positive bacteria. It may be concluded that the chromium(III) complexes used in the present work inhibit the growth of bacteria to a greater extent compared to ciprofloxacin, the reference drug. This fact contrasts with what was found for others chromium(III) complexes containing amino acids as a ligands against Gram negative and Gram positive bacteria, where the MIC values of the complexes are higher than the values for the standard antibiotic [26].

The efficiencies of the ligands and the chromium(III) complexes have been tested against Gram positive, *S. aureus*, and Gram negative, *E. coli*, microorganisms. It is not clear why the other compounds tested, [Ru(phen)_3_]^2+^ and [Ru(bpy)_3_]^2+^, did not show antimicrobial activity at the concentration range used. The results are presented in Table 1. The antimicrobial activity of CrCl_3_ · 6H_2_O has also been investigated. It has been found that CrCl_3_ · 6H_2_O does not exhibit antimicrobial activity at the concentration range used to assay the activity of the complexes in this work. 

In general, for metal complexes showing antimicrobial activity, the following five principal factors should be considered: (i) the chelate effect, that is, ligands that are bound to metal ions in a bidentate fashion, such as the nitrogen-donor ligands phen and bpy, show higher antimicrobial efficiency towards complexes with unidentate nitrogen-donor ligands, for example, pyridine; (ii) the nature of the ligands; (iii) the total charge of the complex, generally the antimicrobial efficiency decreases in the order cationic > neutral > anionic complex, (iv) the nature of the ion neutralizing the ionic complex; (v) the nuclearity of the metal center in the complex; dinuclear centers are usually more active than mononuclear ones [36]. The first two of the five above-mentioned factors are present in our compounds, that is, the chelate effect provided by nitrogen-donor ligands (phen, bpy, and dppz) and the nature of the ligands.

It is evident (Table 1) that the antimicrobial activity of the chromium(III) complexes against *S. aureus* and *E. coli* is better than that of the ligands. A similar behavior was observed by Efthimiadou et al. for copper(II) complexes with nitrogen-donor heterocyclic ligands against *P. aeruginosa*,* S. aureus,* and *E. coli *[37]. Considering the nature of the nitrogen-donor heterocyclic ligand in the chromium(III) complexes, our results suggest that the inhibition of the growth of microorganisms increases in the order dppz > phen, where the [Cr(phen)_2_(dppz)]^3+^ complex was found to be the most potent. 

Finally, when ciprofloxacin was combined with [Cr(phen)_3_]^3+^ for the inhibition of *S. aureus* and *E. coli*, an important synergistic effect was observed (Tables 2 and 3). We found that *S. aureus* was more resistant than *E. coli* to generation of ROS by both antimicrobial agents combined, ciprofloxacin and [Cr(phen)_3_]^3+^, this would be related to less increase of ROS in Gram positive bacteria (Figures 4 and 5).

The present study provides the first evidence of antimicrobial activity of the synthetic metallomolecules [Cr(phen)_3_]^3+^ and [Cr(phen)_2_(dppz)]^3+^. We have synthesized and characterized various *α*-diimine chromium(III) complexes with different ligands, and similar compounds of copper(II) have been recently synthesized, some of which may show a higher degree of antimicrobial activity [38]. Therefore, future studies will be directed to the antimicrobial activity of the newly synthesized complexes, as well as the activity of metal complexes on drug-resistant pathogenic bacteria.

## Figures and Tables

**Table 1:** Minimum inhibitory concentrations (MICs) shown by complexes and ligands against test bacteria.

	MIC in *µ*g/mL (mmol/L)	
	*Staphylococcus aureus *	*Escherichia coli *
[Cr(phen)_3_]^3+^	1 (0.0011)	0.25 (0.0003)
[Cr(phen)_2_(dppz)]^3+^	0.5 (0.0004)	0.125 (0.0001)
CrCl_3_·6 H_2_O	No activity	No activity
[Ru(phen)_3_]^2+^	>1024 (>1.437)	>1024 (>1.437)
[Ru(bpy)_3_]^2+^	>1024 (>1.599)	>1024 (>1.599)
Dipyrido[3,2-a:2′,3′-c]-phenazine	No activity	No activity
1,10-Phenanthroline	No activity	No activity
2,2′-Bipyridine	No activity	No activity
Ciprofloxacin	0.5 (0.0013)	0.125 (0.0003)

**Table 2:** Minimum inhibitory concentrations (MICs) in *µ*g/mL and fractional inhibitory concentration indices (FICIs) of [Cr(phen)_3_]^3+^ in combination with ciprofloxacin against *Staphylococcus aureus* ATCC 29213*.

	Ciprofloxacin (MIC = 0.5)		
[Cr(phen)_3_]^3+^ (MIC = 1)		MIC_A−C_	FIC
MIC_C−A_	FIC	0.033	0.066
0.5	0.5	FICI_C+A_ = 0.566	
0.25	0.25	FICI_C+A_ = 0.316	
0.125	0.125	FICI_C+A_ = 0.191	

*MIC_C−A_: MIC of [Cr(phen)_3_]^3+^ in combination with ciprofloxacin. MIC_A−C_: MIC of ciprofloxacin in combination with [Cr(phen)_3_]^3+^. FIC: fractional inhibitory concentration. FICI_C+A_: fractional inhibitory concentration index (FIC of [Cr(phen)_3_]^3+^ plus FIC of ciprofloxacin).

**Table 3:** Minimum inhibitory concentrations (MICs) in *µ*g/mL and fractional inhibitory concentration indices (FICIs) of [Cr(phen)_3_]^3+^ in combination with ciprofloxacin against *Escherichia coli* ATCC 25922*.

	Ciprofloxacin (MIC = 0.25)		
[Cr(phen)_3_]^3+^ (MIC = 0.125)		MIC_A−C_	FIC
MIC_C−A_	FIC	0.016	0.064
0.064	0.512	FICI_C+A_ = 0.576	
0.032	0.256	FICI_C+A_ = 0.320	
0.016	0.128	FICI_C+A_ = 0.192	
0.008	0.064	FICI_C+A_ = 0.128	
0.004	0.032	FICI_C+A_ = 0.096	

*MIC_C−A_: MIC of [Cr(phen)_3_]^3+^ in combination with ciprofloxacin. MIC_A−C_: MIC of ciprofloxacin in combination with [Cr(phen)_3_]^3+^. FIC: fractional inhibitory concentration. FICI_C+A_: fractional inhibitory concentration index (FIC of [Cr(phen)_3_]^3+^ plus FIC of ciprofloxacin).
